# Crises in Antimicrobial Stewardship: Misuse of Clarithromycin for *Helicobacter pylori* Therapy

**DOI:** 10.3390/pharma3010006

**Published:** 2024-02-20

**Authors:** David Y. Graham

**Affiliations:** Department of Medicine, Michael E. DeBakey Veterans Affairs Medical Center, Baylor College of Medicine Houston, 2002 Holcombe Blvd (111D), Houston, TX 77030, USA

**Keywords:** clarithromycin, antimicrobial stewardship, *Helicobacter pylori*, therapy, vonoprazan, antibiotic misuse, concomitant therapy, Food and Drug Administration, Centers for Medicare and Medicaid Services, Executive Order on Combating Antibiotic-Resistant Bacteria

## Abstract

*Helicobacter pylori* is a class I carcinogen that infects more than 100 million individuals in the United States. Antimicrobial therapy for *H. pylori* has typically been prescribed empirically rather than based on susceptibility testing. Until recently, therapeutic recommendations have generally ignored the principles of antibiotic stewardship. A combination of a proton pump inhibitor (PPI), amoxicillin, and clarithromycin (triple therapy) remains popular despite increasing clarithromycin resistance and poor cure rates. Concomitant therapy (a PPI, amoxicillin, clarithromycin, and metronidazole) is recommended and widely used despite all patients receiving at least one unneeded antibiotic. In 2020, the Food and Drug Administration approved vonoprazan, amoxicillin, and clarithromycin triple therapy, which administers unneeded clarithromycin to >90% of patients (i.e., ~6 tons of unneeded clarithromycin/million treatments). In the late 1980s, the infectious disease community functionally transferred responsibility for the management of *H. pylori* to gastroenterology, which has managed the infection as another common gastrointestinal disease such as constipation. In 2022, both traditional and noninvasive molecular-based susceptibility testing for *H. pylori* became available in the United States. In order to reduce and prevent antibiotic misuse, the infectious disease community should reclaim responsibility for the management of this important infectious disease.

## 1. Introduction

*Helicobacter pylori* infects approximately one-half of the world’s population and causes gastritis/gastric atrophy that may result in peptic ulcer disease and/or gastric cancer [1]. The problems of providing effective *H. pylori* therapy are not currently being embraced by the infectious disease community. For example, triple therapy that combines clarithromycin, amoxicillin, and an antisecretory drug called clarithromycin remains the most used regimen despite generally poor cure rates [2-5]. The regimen was approved by the U.S. Food and Drug Administration (FDA) in 1996, at which time the FDA’s focus was on ulcer healing and recurrence rather than on the treatment of a new infectious disease [6-8]. Because the clinical manifestations were gastrointestinal and obtaining cultures required gastroscopy, this has led to the functional transfer of *H. pylori* from infectious disease to gastroenterology, which has managed the disease as any another common GI disease [9] and without employing the principles of antibiotic stewardship [10-12].

Antimicrobial resistance among *H. pylori* rapidly increased such that clarithromycin, nitroimidazoles, and fluoroquinolones are no longer effective when given empirically [13]. Nonetheless, clarithromycin-containing triple therapy remains the most widely used anti-*H. pylori* regimen worldwide [14,15]. Gastroenterology considered the unavailability of susceptibility testing as unsolvable, requiring “work arounds”. One approach was to add another antibiotic or antibiotics to current regimens to produce multi-antibiotic clarithromycin-containing regimens (e.g., sequential, hybrid, and concomitant therapies) (reviewed in [12]). The most successful regimen was concomitant therapy [16], which is equivalent to the simultaneous administration of clarithromycin and metronidazole triple therapies. Concomitant therapy failure requires resistance to both metronidazole and clarithromycin [17]. However, all patients receive at least one unnecessary antibiotic (Table 1) [18,19].

Table 1 shows the number of ineffective or unnecessary antibiotics used by a population of patients similar to that seen in the United States, with an H. pylori resistance pattern of 20% resistant to clarithromycin, 40% resistant to metronidazole (8% dual resistance), that receives concomitant therapy of a four-drug therapy with a PPI, amoxicillin, clarithromycin, and metronidazole. All patients receive at least one unnecessary antibiotic (either clarithromycin, metronidazole, or both) irrespective of the proportions of antibiotic resistance. From [18], with permission.

The FDA recently approved vonoprazan–clarithromycin–amoxicillin triple therapy in which more than 90% of patients receive clarithromycin unnecessarily [20]. Vonoprazan is a potassium-competitive acid blocker (P-CAB), more potent than traditional PPIs. The effectiveness of vonoprazan triple therapy is driven by the success of the dual P-CAB plus amoxicillin component [20,21].

## 2. Effect of Clarithromycin Resistance on Treatment Success

**Figure 1.**
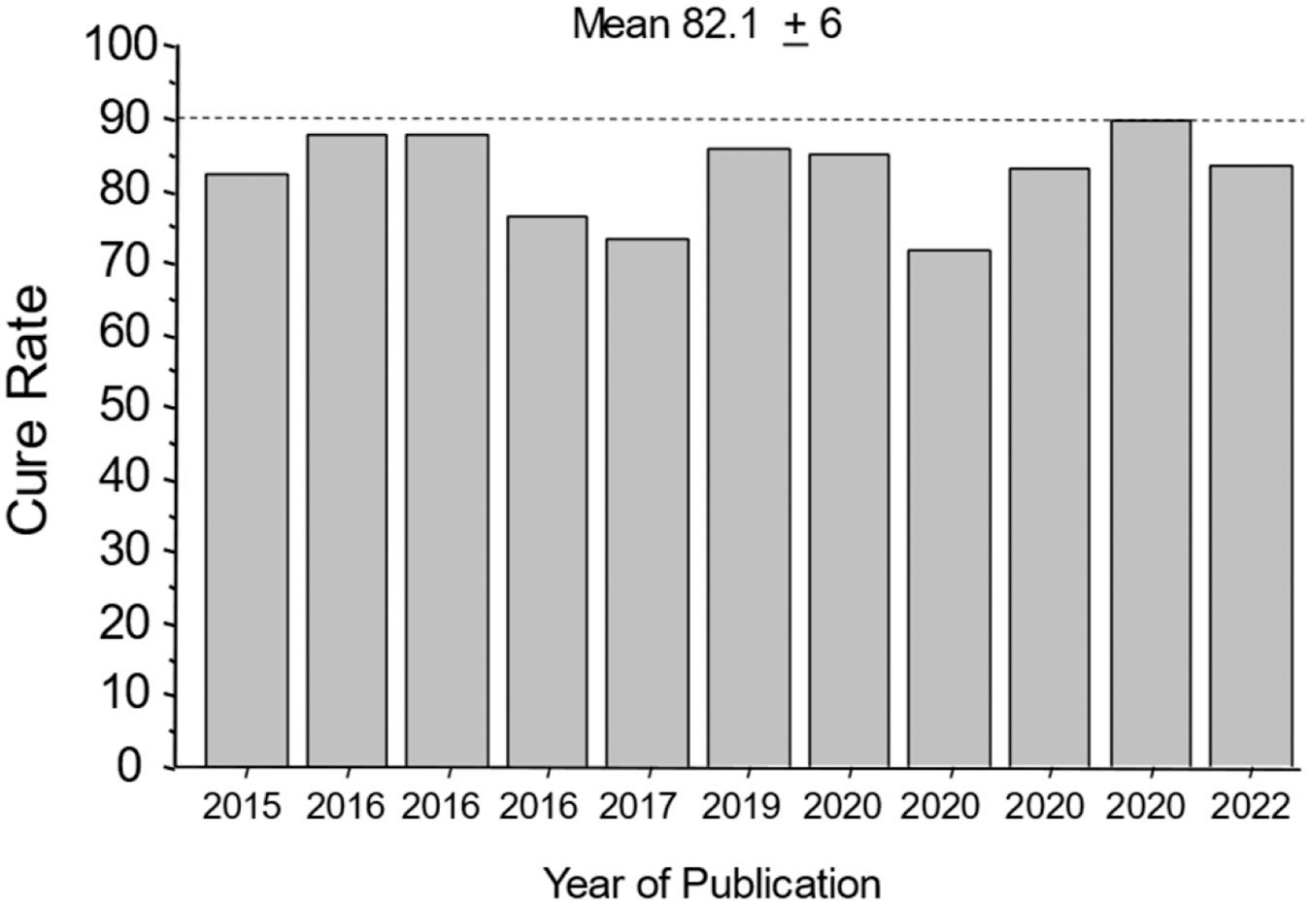
Cure rates with 7-day vonoprazan, clarithromycin, and amoxicillin therapy in the presence of clarithromycin-resistant infections in Japan [36,40-50]. This illustrates the high proportion of patients cured while functionally receiving only the amoxicillin.

Clarithromycin resistance is considered “all or none”, such that clarithromycin resistance reduces clarithromycin triple therapy effectively to just amoxicillin and the antisecretory drug. This results in resistant infections receiving a PPI or P-CAB plus amoxicillin dual therapy and susceptible infections receiving PPI or P-CAB plus amoxicillin and clarithromycin. The results are visualized using an *H. pylori* nomogram (Figure 1) [22].

High *H. pylori* cure rates with amoxicillin require the antisecretory drug to achieve and sustain a high pH [23]. Effectiveness is thus positively related to the potency of the antisecretory drug and inversely correlated with the ability of the stomach to make acid. PPIs’ relative potency is assessed as the proportion of a 24-h day during which the pH is maintained at pH 4 or above (pH4 time) [24]. Pantoprazole, omeprazole, and lansoprazole are rapidly metabolized by CYP2C19, which reduces their effectiveness in populations where rapid metabolizers are common (e.g., most of the Western world). Rabeprazole and esomeprazole are the most potent PPIs and are minimally affected by CYP2C19. They are preferred PPIs for *H. pylori* therapy [25].

Amoxicillin dual therapy using traditional PPIs is unable to reliably cure a high proportion of cases in Western populations. In contrast, rabeprazole or esomeprazole dual therapies have often proved effective in Japan and in some Chinese populations [26-35].

## 3. Potassium-Competitive Acid Blockers (P-CABs)

The effectiveness of vonoprazan plus antibiotics for the treatment of *H. pylori* infections was first demonstrated in Japan [36]. The Japanese pivotal clinical study compared b.i.d dosing of a low potency PPI (lansoprazole 30 mg; equivalent to 27 mg omeprazole) [24] with 20 mg of vonoprazan (approximately equivalent to >40 mg of esomeprazole b.i.d.), each given b.i.d. for the cure of *H. pylori* infections [36]. With clarithromycin-susceptible infections, both the vonoprazan- and PPI-containing triple therapies produced high (~97%) and near-identical cure rates [36]. However, in the presence of clarithromycin resistance, the regimens were reduced to the antisecretory drug plus amoxicillin. The cure rates of the dual therapies were therefore dependent on the relative antisecretory potency of the antisecretory drug (i.e., the cure rate was 40% with lansoprazole vs. 82% with the more potent vonoprazan) [21]. The overall high cure rate obtained (82%) with clarithromycin resistance was consistent with the notion that clarithromycin was responsible for only a small proportion of those cured (i.e., 82% would have been cured without clarithromycin) [21]. It was also noted that the optimization of the vonoprazan–amoxicillin dual therapy should produce a highly effective dual therapy without the need for clarithromycin. However, vonoprazan triple therapy was introduced despite more than 80% receiving unneeded clarithromycin. Vonoprazan–clarithromycin triple therapy is currently the most widely used treatment of *H. pylori* in Japan with more than 1.5 million patients treated yearly [37]. However, over time, the overall cure rates have fallen closer to 80% as clarithromycin resistance has continued to increase [38]. In the U.S./European trial, the cure rates for both clarithromycin-susceptible and resistant strains were unacceptably low and the proportion receiving unneeded clarithromycin was higher (>90%) (see below) [39]. Despite the poor outcomes, both vonoprazan triple and dual therapies were approved in the United States and Europe, although neither has yet been released due to manufacturing issues.

## 4. Unnecessary Clarithromycin Resulting from Vonoprazan Triple Therapy

The results of the Japanese pivotal study (high success with the amoxicillin dual therapy and a high rate of unnecessary clarithromycin) have been confirmed by 11 vonoprazan triple therapy studies that provide cure rates in relation to clarithromycin resistance (i.e., the success is attributable to the amoxicillin) [36,40-50] (Figure 1).

**Figure 2.**
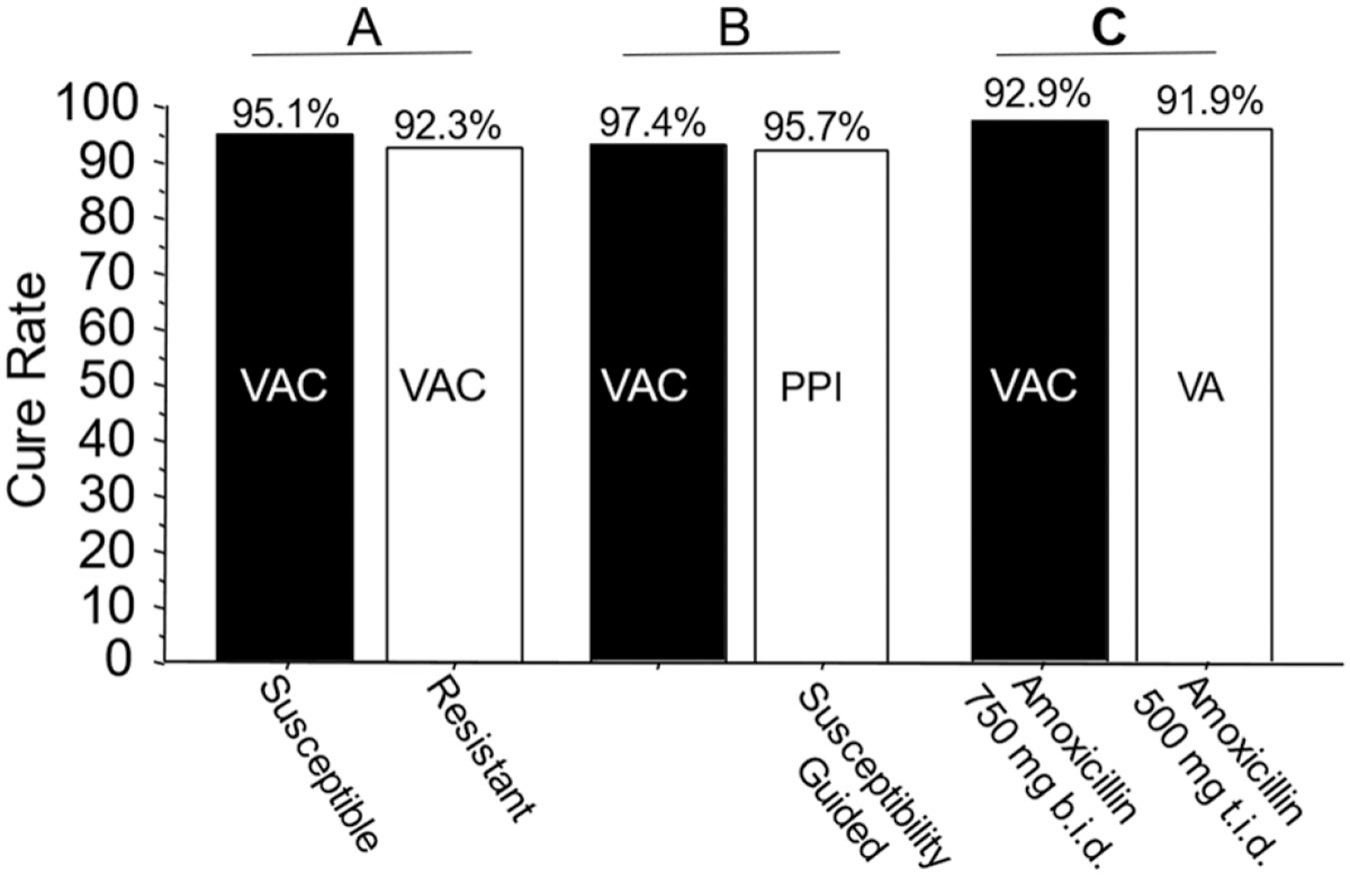
Examples of comparative studies in Japan. Legend: Panel A: Cure rates of 7-day vonoprazan, clarithromycin, and amoxicillin therapy in clarithromycin-susceptible and resistant infections in Japan. The resistant infections are functionally seen in vonoprazan and amoxicillin dual therapy [50]. Panel B: Comparison of the effectiveness of vonoprazan–clarithromycin triple therapy vs. susceptibility-based PPI clarithromycin triple therapy in the same population [52]. Panel C: Comparison of 7-day vonoprazan triple and dual therapy in the same population. In the triple therapy, 1500 mg of amoxicillin was given as 750 b.i.d. vs. 500 mg t.i.d. in the dual therapy [53]. VAC—vonoprazan, amoxicillin, clarithromycin; PPI AC—PPI, amoxicillin, clarithromycin; VA—vonoprazan, amoxicillin. Vonoprazan was 20 mg b.i.d., clarithromycin was 200 mg b.i.d., amoxicillin was 750 mg b.i.d. except where shown.

The mean cure rate attributable to amoxicillin dual therapy with a 7-day vonoprazan triple therapy was 81.7 ± 6% [36,40-49,51]. A second approach to quantitate the proportion receiving unnecessary clarithromycin was to examine the cure rates of dual therapy (which functionally contains no clarithromycin) vs. triple therapy with clarithromycin in the same populations (Figure 2A) [50]. These different comparisons all confirmed that the majority of the clarithromycin given in vonoprazan triple therapy was unneeded.

**Figure 3.**
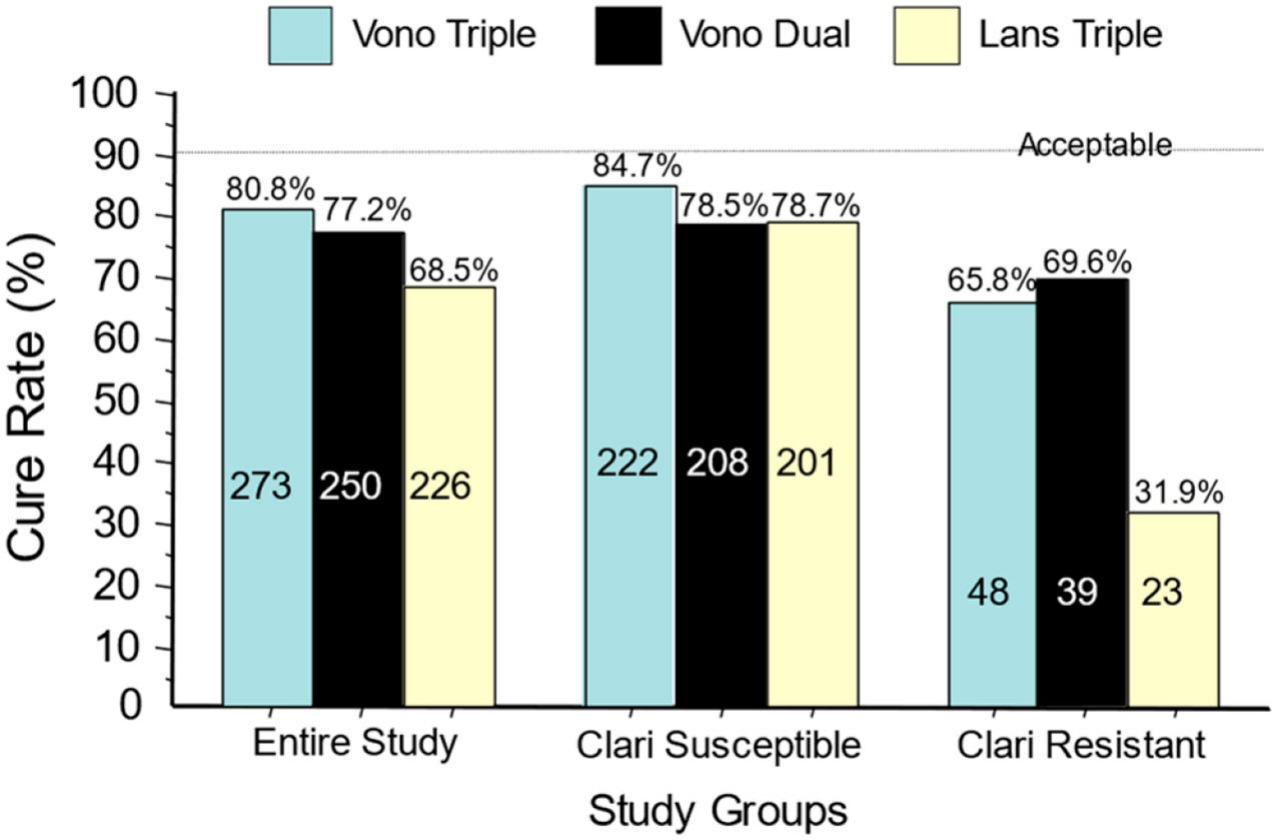
Cure rates for the U.S./European vonoprazan clinical trial of vonoprazan, clarithromycin, and amoxicillin; vonoprazan plus amoxicillin dual therapy; and lansoprazole–clarithromycin triple therapy [55]. The data are shown for the entire group and separately for those with clarithromycin-susceptible infections and those with clarithromycin-resistant infections. No arm achieved the expected cure rate of >90%. The number of subjects in each are shown in the columns (from reference [20], with permission).

The U.S./European vonoprazan–amoxicillin–clarithromycin clinical trial differed from the Japanese trials in dosing, duration, and effectiveness. Despite higher doses of antibiotics and doubling the duration from 7 to 14 days, both the U.S./European study and a study in Thailand yielded relatively poor cure rates compared to Japan, even with susceptible strains (e.g., 85.7% with the P-CAB) [39,54]. This was both unexpected and unprecedented [20,55] (Figure 3).

**Figure 4.**
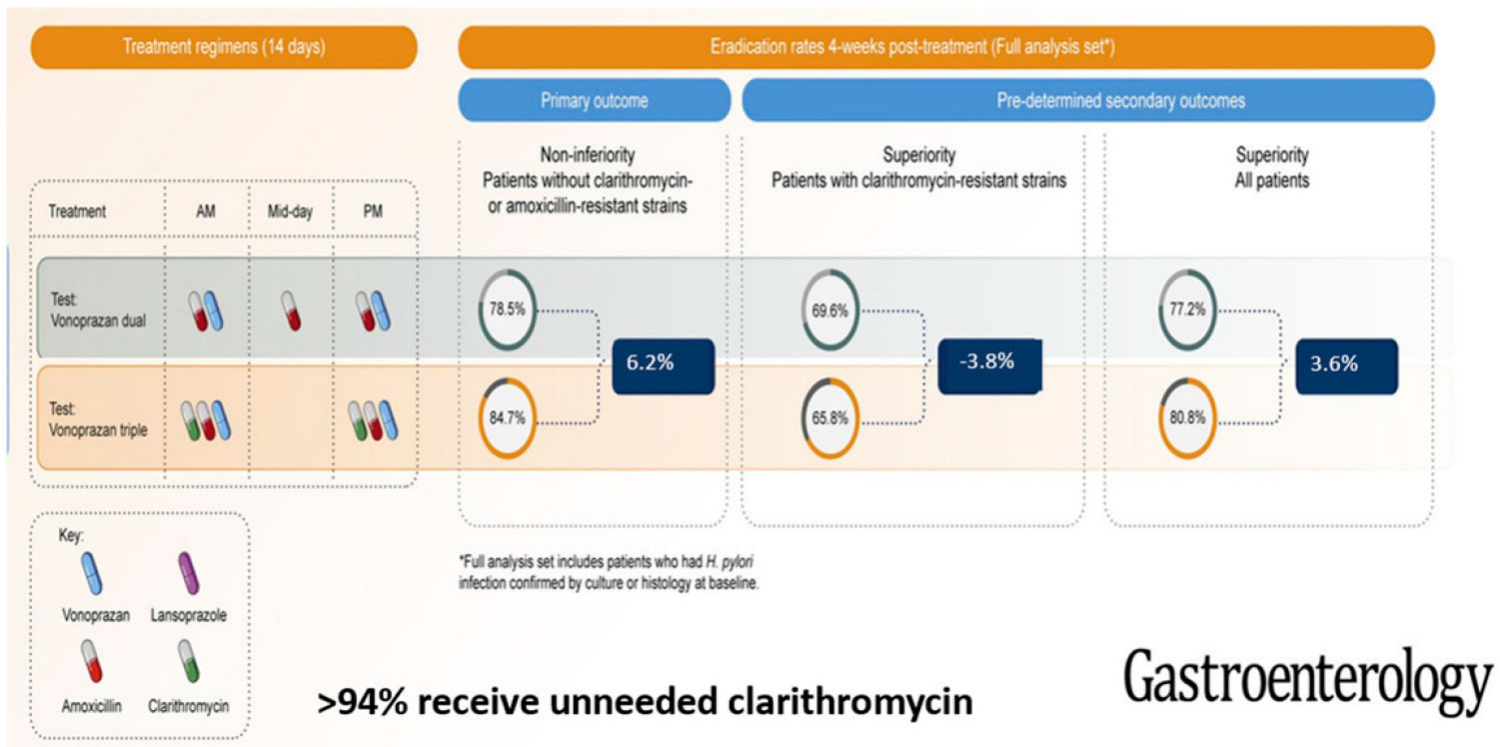
Results of the U.S./European vonoprazan clinical trial graphical abstract, adapted from [39], limited to the comparison of the vonoprazan triple and dual arms. Modification consisted of subtraction of the results with vonoprazan dual therapy from the results with triple therapy to show the relative benefits of adding clarithromycin. The vonoprazan dual therapy contained 3 g of amoxicillin vs. 2 g for the triple therapy, which also contained clarithromycin. The maximum difference in those with susceptible strains was 6.2%, showing that >93% received no antimicrobial benefits from the presence of clarithromycin.

The cure rate with susceptible strains using amoxicillin b.i.d. was 6.2% greater than dual therapy with amoxicillin t.i.d. The proportion receiving unnecessary clarithromycin was also evaluated both in relation to the differences in cure rates with susceptible vs. resistant infections and with dual vs. triple therapies (Figures 3 and 4) [20].

These differences ranged between 3% and 6%, which is consistent with the notion that more than 90% of individuals received clarithromycin unnecessarily [20,39] (Figure 4). Despite the high proportion receiving unneeded clarithromycin, vonoprazan–clarithromycin triple therapy received FDA approval without restrictions [55].

## 5. Quantifying the Amount of Unnecessary Clarithromycin Used with Vonoprazan Triple Therapy

In Japan, the quantity of clarithromycin used per million cases treated using either the 200 or 400 mg b.i.d. approved dosages of clarithromycin (i.e., dose times duration times the number participants) would be, respectively, 2800 kg and 5600 kg. In the United States and Europe, where the clarithromycin dosage is 1 g/day for 14 days, the results per million treated is equivalent to 14,000 kg/year or 6.35 tons/year/million treated. In both instances, 80% to 90% of the clarithromycin would be unnecessary (i.e., misused). As noted above and evident in Figure 4, optimizing the vonoprazan–amoxicillin dual therapy should achieve equivalent or superior results without the need for clarithromycin.

## 6. FDA Approval of Vonoprazan Triple and Dual Therapy despite Poor Cure Rates and the Unnecessary Administration of Clarithromycin

As noted above, until recently, the FDA’s focus was on ulcer healing and only patients with ulcers or proven ulcer disease were allowed in treatment trials for FDA approval [56]. In the initial FDA trials, cure rates were often low [6] (e.g., rabeprazole, the last PPI-based triple therapy approved, achieved a cure rate of only 71%) [57]. In the most recent example (U.S./European vonoprazan trial), cure rates were poor overall (80%) and were only 85% for susceptible infections (Figure 3) [55].

Traditionally, with other antibiotics, FDA approval is only attempted after the proposed regimen has undergone intensive testing and optimization. Approval of poorly performing unoptimized *H. pylori* regimens presents a problem for clinicians wishing to provide effective therapy. Other examples of unoptimized regimens obtaining approval include Pylera and Talecia, neither of which has subsequently been optimized. Expecting optimization by the pharmaceutical company after FDA approval is likely to be unrealistic, as the costs of repeating the study would be prohibitive. In addition, *H. pylori* combination therapies are generally prepackaged for per-patient use, making it difficult or impossible for clinicians to obtain the drugs separately to preform optimization. Also, when the drugs are co-packaged, physicians cannot easily alter the dosage or durations, even if the approved regimen differs from what is subsequently shown to be the optimal combination. The lack of optimization prior to approval and the FDA’s willingness to approve unoptimized, generally poorly performing *H. pylori* therapies has allowed drug companies to rank marketing ahead of effectiveness. An example is Pylera, a prepackaged 10-day combination bismuth quadruple therapy; 14-day therapy is currently recommended [13]. In Western countries, bismuth subcitrate is generally not available except as part of Pylera. To achieve a 14-day therapy would require the purchase of very expensive prepackaged medication [58,59]. Going forward, the optimization of *H. pylori* treatment regimens should be a requirement before submission to the FDA. However, optimization may still need to be repeated in new populations as it is currently not possible to predict outcomes based on experience in different populations or countries. As noted, the FDA-approved vonoprazan combinations differ markedly from those used in Japan in terms of duration and amoxicillin and clarithromycin dosing. Fundamentally, the company had to guess regarding the optimum composition for the U.S./European populations, and that guess proved to be a bad one. We believe that the approval of vonoprazan–clarithromycin combinations should either be rescinded or the misuse of thousands of kilograms of clarithromycin per year justified.

## 7. The Future of *H. pylori* Therapy: Vonoprazan Dual Therapy

PPI–amoxicillin dual therapy was introduced by Peter Unge in 1989 [60], only later to be largely abandoned because of the inability to reliably achieve sufficient suppression of acid secretion, except in Asia, and even there, the results with first generation PPIs were not reproducible [11,61,62]. The relatively high cure rates obtained with vonoprazan in the presence of clarithromycin resistance (the surrogate for dual therapy) rekindled interest in dual therapy. As discussed above, there are numerous trials and meta-analyses investigating the use of different PPIs, PPI dosages, durations, and amoxicillin dosage and frequency of administration in dual therapy. Studies of the duration of therapy (i.e., 7, 10, or 14 or more days) have favored longer durations (e.g., [31,33]). The optimal amoxicillin dosage remains unclear, as high cure rates have been achieved with a wide range of dosing, ranging from 1 to 3 or more grams daily; Both good and poor results have been reported with this variation in dosages. Considerations for the optimization of P-CAB-containing dual therapy include increasing vonoprazan dosage [63] and increasing the duration of treatment or the use of adjuvants to enhance the pH effect. Examples of adjuvants include the addition of a PPI, H2-receptor antagonist [64], an anticholinergic [65], and/or even antacids [20]. Studies with higher vonoprazan doses (e.g., 60 mg b.i.d.) in Australia have provided the highest cure rates [63]. Patient characteristics, especially location (Asia vs. the United States), are currently the best predictors of outcome. Body size (body surface area and BMI) reduces the effectiveness of P-CABs, whereas the presence and severity of corpus gastritis improve treatment outcomes [66]. Overall, highly effective dual therapy requires excellent control of gastric pH, suggesting that optimization will often be required for different populations (i.e., one size may not fit all) [35,46,53,67-76].

## 8. Failure of Governmental Oversight of Antimicrobial Stewardship and Antibiotic Misuse in the Treatment of *H. pylori* Infections

The management of *H. pylori* infections has largely been within the purview of gastroenterology, resulting in therapeutic recommendations and guidelines that have more often been opinion-based rather than susceptibility-based. For 40 years, gastroenterologists have unsuccessfully attempted to manage an infectious disease without susceptibility testing. During this same period, the infectious disease community, the World Health Organization, and the United States Government have increasingly focused on antimicrobial resistance and antibiotic stewardship. In 2014, President Barack Obama issued Executive Order 13,676 regarding Combating Antibiotic-Resistant Bacteria. This order established the Task Force for Combating Antibiotic-Resistant Bacteria, which outlined five interrelated goals to guide Federal action [77,78]. The executive order was followed by The National Action Plan for Combating Antibiotic-Resistant Bacteria 2020–2025 [77]. In 2019, the Centers for Medicare and Medicaid Services (CMS) issued a rule requiring all hospitals participating in CMS programs to establish antimicrobial stewardship programs by 30 March 2020 [79,80]. These orders and regulations focused on antibiotic stewardship and included requirements for the optimization of antimicrobial therapy, which included drugs, formulations, doses, and dosing intervals. It also required obtaining and updating local, regional, and national susceptibility data to provide regularly updated guidance regarding diagnosis and therapy. The CDC documents specifically required the creation and promotion of evidence- and susceptibility-based treatment guidelines, tracking of antibiotic dispensing, and setting targets for improvement (i.e., monitoring and reporting).

As far as the diagnosis and treatment of *H. pylori* is concerned, these events never happened. One reason may be that the focus of the requirements is on hospitals and academic experts in laboratory medicine and infectious diseases who may not recognize that *H. pylori* should be included. The Veterans Affairs Hospital System, which has a high prevalence of *H. pylori*-infected patients, was also unaffected, and thus missed the opportunity to lead the nation in relation to the management of *H. pylori* infections. *H. pylori* has largely remained under the radar, despite being present in at least one-third of American citizens and the majority of recent immigrants from Mexico and Central and South America. It is responsible for many, if not most, cases of atrophic gastritis and most cases of upper gastrointestinal bleeding seen in those same hospitals, as well as essentially all gastric cancers. For 40 years, gastroenterologists have tried to manage this infectious disease without susceptibility testing and have failed, and even the FDA treats *H. pylori* as if the rules, practices, and regulations pertaining to infectious diseases do not apply.

In a 2020 article in GI Hepatology News, we, Hashem El-Serag and I, recommended that organized gastroenterology join with the infectious disease community to make *H. pylori* an infection of joint interest [81]. We hypothesized that the CMS rule would ultimately result in significant changes in the approach to treating *H. pylori* infections that included improved testing and availability and implementation of knowledge of local susceptibility and resistance patterns. We were wrong! *H. pylori* has largely remained invisible to hospital culture laboratories, to those responsible for implementing the CMS rules, and those charged with preventing the misuse of antibiotics.

In 2022, a number of major reference laboratories, possibly influenced by the CMS rules, began offer culture and susceptibility testing [82]. More importantly, noninvasive susceptibility testing of *H. pylori* using stools has also become available using next-generation sequencing for the six commonly used antibiotics, as well as for fresh or previously obtained gastric mucosal biopsies contained in paraffin blocks [82,83]. Susceptibility testing of stools for clarithromycin using the polymerase chain reaction (PCR) also became available from Mayo Clinic Laboratories [82,83]. However, in response to the CMS rule, few, if any, hospital laboratories have begun to offer routine susceptibility testing and even fewer have welcomed or made these services available as a “send out”. Susceptibility testing by culture and by next-generation sequencing for *H. pylori* is still not reimbursed by Medicare or most insurance companies.

The President’s order set deadlines for establishing new antimicrobial stewardship programs and CMS provided details for implementing the rules, but, somehow, *H. pylori* appears to have been left out. The order to track antibiotic dispensing and the setting of targets for improvement (i.e., monitoring and reporting) in relation to *H. pylori* apparently fell on deaf ears. As noted above, the poorly performing clarithromycin triple therapy remains the most commonly prescribed regimen [84]. The order to track and manage antibiotic dispensing for *H. pylori* has either gone unheeded or the information has not been acted upon. Clearly, hospitals and other culture laboratories, pharmacies, and infectious disease specialists who oversee antibiotic use in groups or hospitals must, as directed by the Executive Order on Combating Antibiotic-Resistant Bacteria, the CDC, and CMS, become involved. Therapies that reliably cure *H. pylori* without unnecessary antibiotics should be approved and used. The use of regimens that fail to reliably achieve high cure rates must be abandoned. Although it was impossible to foresee that the FDA would approve a regimen with a relatively low cure rate in which the majority of patients received unnecessary antibiotics (i.e., vonoprazan triple therapy), that mistake can be rectified.

We previously recommended establishing quality metrics related to appropriate diagnostic testing for both the initial infection and post-treatment evaluations [81]. The 2020 CMS rule provided both the impetus and the methods to move forward and deal with *H. pylori* like other infectious diseases. However, to date, it has had little effect on *H. pylori*. It is time to enlist our colleagues in infectious disease to become involved and include *H. pylori* as one of their diseases.

## Figures and Tables

**Table 1.** Hypothetical scenario of number of unnecessary drugs for *H. pylori* therapy depending on antibiotic sensitivity patterns.

Susceptibility Pattern of *H. pylori* to Clarithromycin Susceptible 80%; Resistant 20%	Clarithromycin and Metronidazole Susceptible 60%	Prevalence of Pattern	Successful Treatment	Number Ineffective	Number Unnecessary Drugs Used
Susceptible	Susceptible	48%	Yes	0	1
Susceptible	Resistant	32%	Yes	1	1
Resistant	Susceptible	12%	Yes	1	1
Resistant	Resistant	8%	No	2	2
